# The influence of health-risk perception and distress on reactions to low-level chemical exposure

**DOI:** 10.3389/fpsyg.2013.00816

**Published:** 2013-11-05

**Authors:** Linus Andersson, Anna-Sara Claeson, Lisa Ledin, Frida Wisting, Steven Nordin

**Affiliations:** Department of Psychology, Umeå UniversityUmeå, Sweden

**Keywords:** health-risk perception, olfaction, environmental psychology, perception, sensitization, bias, distress, cognition

## Abstract

The general aim of the current study was to investigate how perceived health risk of a chemical exposure and self-reported distress are related to perceived odor intensity and odor valence, symptoms, cognitive performance over time as well as reactions to blank exposure. Based on ratings of general distress, 20 participants constituted a relatively low distress group, and 20 other participants a relatively high distress group. Health risk perception was manipulated by providing positively and negatively biased information regarding *n*-butanol. Participants made repeated ratings of intensity, valence and symptoms and performed cognitive tasks while exposed to 4.7 ppm *n*-butanol for 60 min (first 10 min were blank exposure) inside an exposure chamber. Ratings by the positive and negative bias groups suggest that the manipulation influenced perceived health risk of the exposure. The high distress group did not habituate to the exposure in terms of intensity when receiving negative information, but did so when receiving positive information. The high distress group, compared with the low distress group, rated the exposure as significantly more unpleasant, reported greater symptoms and performed worse on a cognitively demanding task over time. The positive bias group and high distress group rated blank exposure as more intense. The main findings suggest that relatively distressed individuals are negatively affected by exposures to a greater degree than non-distressed.

## Introduction

In a series of seminal studies, Dalton and colleagues showed that the words used to describe a chemical significantly alters how individuals react when being exposed to it. Exposure described as harmful elicited higher ratings of intensity and sensory irritation over time, compared with identical exposure described in a positive or neutral fashion. Moreover, individuals receiving negative rather than positive or neutral information reported more symptoms after an exposure session (Dalton, 1996, 1999; Dalton et al., 1997). Dalton and colleagues utilized an exposure chamber, but similar effects have been found when using transient stimuli. Djordjevic et al. (2008) found that negative, compared with positive or neutral odor labels, result in significantly higher intensity ratings and lower ratings of pleasantness of odorants delivered in glass bottles. Ratings of hedonic value, argued to be the dominant dimension in olfaction (Richardson and Zucco, 1989), seems to be more easily influenced by differently phrased information than ratings of intensity (Djordjevic et al., 2008; Nordin et al., 2013). Providing differently phrased information about an exposure does not always seem to influence intensity ratings (Kobayashi et al., 2007), and the effect seems to be greater for chemicals eliciting trigeminal sensations (i.e., pungency; Dalton et al., 1997).

Nevertheless, the outcomes of these studies show that neither the perceived properties of an airborne chemical, nor its assumed health effects depend solely on the type and strength of the exposure. If the results are applicable outside the laboratory, they suggest that expectancy of possible health risks is a factor to consider when evaluating and setting exposure limits. This argument is corroborated by population-based studies emphasizing the importance of health-risk perception as an indicator of symptom reports (Shusterman et al., 1991; Claeson et al., 2013). Indeed, no exposure is actually necessary for people to report symptoms attributed to chemicals, as shown by sham exposure studies (Knasko et al., 1990; Lange and Fleming, 2005). In addition to the sensory and hedonic aspects reviewed above, Nordin et al. (2013) reported that negative health-risk perception has deleterious consequences for cognitive performance.

Reactions to chemicals are also influenced by the constitution or general well-being of the exposed individual. Negative affectivity is a trait that has been associated with greater unpleasantness ratings and symptom reports after chemical exposure (Dalton, 2002; Smeets and Dalton, 2005). Chen and Dalton (2005) reported that anxious women rated the intensity of both pleasant and unpleasant chemical stimuli as higher than did non-anxious women. Highly anxious, compared with non-anxious women, also report more symptoms when exposed to low levels of chemical solvents (Orbæk et al., 2005). Ihrig et al. (2006) found that positive and negative affectivity influences symptom reports from men as well–an effect most clearly seen with low-level exposure. At higher concentrations the impact of such traits was diminished. Several other traits or conditions associated with higher reactivity to chemical exposures have been reported in the literature, including chemical intolerance (Andersson et al., 2009a, b), migraine (Sjöstrand et al., 2010) as well as neurologic and endocrine disorders (Spielman, 1998).

Situational circumstances and predisposing traits are not only relevant for short-term reactions to chemicals commonly investigated in exposure studies. They constitute two main factors in models of medically unexplained symptoms. In combination, they are assumed to increase the risk of developing long-term illness. Vulnerable individuals confronted with a deleterious exposure is at risk of developing a vicious cycle of responses that is maintained over time (Richardson and Engel, 2004; Deary et al., 2007; McEwen, 2007; Ganzel et al., 2010). The temporal aspect of the findings by Dalton and colleagues (Dalton, 1996, 1999; Dalton et al., 1997) becomes relevant in this context as sensitization (i.e., increased responses over time) can be seen as an indication of an illness generating cycle. For instance, sensitization has been hypothesized to be the characteristic feature of medically unexplained symptoms such as chemical intolerance or chronic pain (Overmier, 2002; Yunus, 2008). Habituation (i.e., decreased responses over time) is the opposite to sensitization. Investigating how situational and predisposing factors interact to generate sensitized responses may be relevant for occupational health issues and can assist in pinpointing individuals at risk of developing clinical conditions.

In this vein, the general aim of the current study was to investigate how health-risk perception, manipulated by biased information, and rated distress are related to sensitization/habituation in individuals exposed to low, non-toxic concentrations of an airborne chemical. Based on the literature reviewed above, our first hypothesis was that individuals reporting relatively high distress would sensitize to a weak chemical exposure described in a negative manner, whereas individuals reporting relatively low distress would habituate. Sensitization/habituation was assessed by ratings of perceived intensity and pleasantness/unpleasantness of the chemical *n*-butanol, as well as symptoms over time. The second hypothesis was that individuals receiving negative information bias and reporting higher distress would perform worse on cognitive tasks during exposure compared with negatively biased individuals reporting lower distress. We also investigated whether information bias and distress were related to a tendency of reacting to blanks, i.e., making false alarms.

## Method

### Participants

Forty non-smoking, non-pregnant participants aged between 18 and 35 years with a self-reported normal sense of smell were recruited through billboard advertisements on Umeå University campus and public areas such as the hospital, library, employment office and cafés. Prior to the exposure, participants were screened for anosmia (constituting an exclusion criterion) using a 0.44% v/v (336 ppm) concentration of n-butanol (99%, Merck) of the Connecticut Chemosensory Clinical Research Center Threshold Test (Cain, 1989).

Subsequent to the exposure, all participants filled out the SCL-90 inventory (Fridell et al., 2002). The SCL-90 is a widely used self-report symptom inventory covering nine symptom dimensions: somatization, obsessive-compulsive, interpersonal sensitivity, depression, anxiety, hostility, phobic anxiety, paranoid ideation, and psychotism (Derogatis et al., 1976). The mean score of all the items of the SCL-90 constitutes the Global Severity Index (GSI) and has been argued to be a good measure of general distress or well-being (Cyr et al., 1985; Fridell et al., 2002). We performed a median split to divide the participants into two groups based on the GSI. Those with a relatively low GSI constituted the low distress group. Those with a relatively high GSI constituted the high distress group. Importantly, the distress groups in this regard refer to non-pathological variations in the population. Descriptive data of the participants are given in Table 1. There was no significant difference between the two bias groups in terms of GSI score, age or sex, as assessed by independent samples *t*-tests and Mann-Whitney *U*-tests (all *t* and *Z* < 0.9; all *p* > 0.38).

**Table 1:** **Descriptive data of the participants, clustered according to distress and bias group**.

	**Positive bias**	**Negative bias**	**Pos and neg bias**
Low distress, *n*	10	10	20
Women / men, *n*	5/5	4/6	9/11
Age, *M* years (±*SD*)	25 (4.2)	24 (3.6)	25 (3.9)
GSI, *M* (±*SD*)	0.19 (0.08)	0.16 (0.07)	0.18 (0.07)
High distress, *n*	10	10	20
Women / men, *n*	5/5	6/4	11/9
Age, *M* years (±*SD*)	24 (4.3)	24 (4.9)	24 (4.5)
GSI, *M* (±*SD*)	0.65 (0.21)	0.64 (0.51)	0.64 (0.38)
Low and high distress, *n*	20	20	40
Women / men, *n*	10/10	10/10	20/20
Age, *M* years (±*SD*)	24 (4.2)	24 (4.2)	24 (4.2)
GSI, *M* (±*SD*)	0.42 (0.27)	0.40 (0.43)	0.41 (0.36)

GSI = Global Severity Index of the SCL-90.

All participants were given written and spoken information about the study. The study was conducted in accordance with the Helsinki Declaration and approved by the Ethics Committee at Umeå University (# 2012-154-31M). A signed informed consent was obtained from each participant. All participants were given 200 SEK (~20 EUR) for their participation.

### Materials and procedures

#### Chemical exposure

Participants were exposed to *n*-butanol (99.4% Baker) at a concentration of 4.7 ppm while seated in a windowed exposure chamber. *n*-Butanol was chosen since it was considered relatively ambiguous and unfamiliar, which was expected to facilitate the information bias manipulation. The concentration was chosen to be clearly detectable (above the olfactory threshold 40 ppb; Nagata, 2003) but well–below the threshold for sensory irritation (24.5 ppm; Ruth, 1986). The intensity was also chosen based on pilot testing. The stimulus material was vaporized using a nebulizer. To ensure a consistent concentration in the exposure chamber a known amount of the odorant was fed through the nebulizer into a feed stream of filtered air monitored by a mass flow controller. The mixture was then diluted (by another stream of filtered air) to the desired concentration before it was fed into the exposure chamber. The vapor-phase concentration was measured inside the exposure chamber with a photoionization detector (PID, RAE Systems). The exposure chamber has a volume of 2.7 m^3^ (height × width × depth: 200 × 90 × 150 cm). Air was exchanged at a rate of 7.8 times per hour. The mean temperature across participants at the end of testing was 22.3°C (*SD* ± 1.0), and the relative humidity was 18.9% (*SD* ± 2.5).

#### Information bias

Participants were given either positive or negative information regarding the chemical used for exposure. The negatively biased group was told that butanol is an industrial solvent that can produce symptoms at higher concentrations, and that the aim of the study was to assess possible negative effects on performance at levels below the toxicological threshold. When seated in the exposure chamber, the negatively biased group could see a poster showing hazard pictograms and risk phrases associated with *n*-butanol. The positively biased group was told that butanol is a natural extract found in many food products, and can be produced by fermenting, e.g., corn. These participants were told that the aim of the study was to investigate whether ambient *n*-butanol could diminish sleepiness, possibly resulting in greater cognitive performance. While seated in the chamber, the positively biased group could see a poster with chocolate bars and a text informing the reader that butanol is an important component in high quality chocolate. The posters were placed on the laboratory wall so that the participants could see them easily, but at such a distance that they did not seem directed to the person sitting in the chamber. The rationale for using the posters was to remind the participants of the biased information during the exposure, in a manner not obviously and suspiciously directed at them. Neither of the bias groups were misled, as both the positive and negative information are in fact true.

#### Apparatus

The sequence of psychophysical ratings and cognitive tasks was programmed using E-Prime 2.0 software (Psychology Software Tools, Pittsburgh, PA). A Windows 7 laptop computer (Compaq 8510) connected to a 24 inch screen (Asus VK246H) in front of the participants and a Microsoft Bluetooth Number Pad placed on a lap tray were used to present tasks and record responses.

### Tasks

#### Ratings of intensity, valence and symptoms

Participants rated the chemosensory intensity and valence of the exposure using a Borg CR-100 scale (Borg and Borg, 2002).The CR-100 is a verbally anchored ratio scale (Borg, 1998; Borg and Borg, 2001) with descriptive adjectives that correspond to specific numbers on the scale: Nothing at all, 0; minimum, 1.5; extremely weak, 2.5; very weak, 6; weak, 12; moderate, 25; strong, 45; very strong, 70; extremely strong, 90; near maximal, 100. Numbers above 100 are not labeled, but approaches the label absolute maximum. For valence ratings the participants were prompted to add a plus sign before the rating if the exposure was judged as pleasant, and a minus sign before the rating if deemed unpleasant.

Ten symptoms were rated using the Borg CR-100 scale (Borg and Borg, 2002). These constituted eye irritation, nasal mucosal irritation, skin irritation, throat irritation, shortness of breath, concentration difficulties, dizziness, tiredness, headache and nausea. They were chosen since they have been shown to frequently (20–69%) be reported by persons with chemical intolerance (Andersson et al., 2009a), and since they together represent a broad range of symptoms (airway, mucosae, skin, cognitive, head-related, and gastrointestinal). The mean of these 10 symptoms were used as a composite score in the statistical analysis.

#### Plus/minus lists

Participants performed a plus/minus task based on Jersild (1927). Participants were prompted to add, subtract or alternate between adding and subtracting three from a random two-digit number ranging from 13 to 96. Each plus/minus list block consisted of one addition list, one subtraction list and one alternating list in which the task was to shift operation after each number. Participants were told to perform the tasks as quickly and correctly as possible. After each input, the screen either flashed green if the answer was correct, or red if incorrect. Each list had the duration of 60 s. The plus/minus lists were assumed to be related to general cognitive performance. The task was chosen based on a study by Nordin et al. (2013) in which biased information influenced the performance of this task. Task performance was analyzed based on the mean number of correct answers in the plus, minus and plus/minus lists within each block.

#### Updating task

Participants performed an additional cognitive task, assumed to be more difficult than the plus/minus lists. It was based on the letter memory task described in Miyake et al. (2000). In the current task, single numbers (1, 2, 3 or 4) were presented serially on the center of the screen for 2000 ms with a 1000 ms inter-stimulus interval. Participants were to recall and type in the last four numbers in the correct order after each list. Seven lists were presented in random order, with a length of 5, 7, 9, 11, 13, and 15 digits. The list length was unknown to the participants. Number of correctly recalled sequences was used as a measure of task performance.

### Procedure

**Figure 1:**
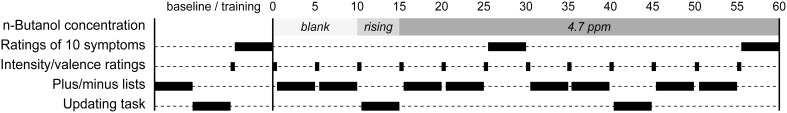
**Overview of experimental procedure.** The exposure session began at min 0.

An overview of the experimental procedure is provided in Figure 1. After giving the informed consent, receiving the biased information and passing the odor detection test, participants were seated in a chair inside the chamber with the door open. The participants received the lap tray with a numerical keyboard through which responses were recorded. Participants practiced the plus/minus lists, the updating task and how to rate intensity and valence. They also rated their baseline symptoms. After the approximately 15 min training/baseline session, participants were informed that the actual study would begin right after the chamber door was closed. They were told that the concentration of the chemical inside the chamber could vary during the session. Unknown to the participants, no chemical was delivered into the chamber during the first 10 min of testing. After the 10 min period of blank exposure, the *n*-butanol was released into the chamber and reached its peak concentration after about 8 min. The concentration remained at this peak level for the rest of the session. During the exposure, participants performed a total of 12 ratings of intensity and valence, eight blocks of plus/minus lists, two blocks of updating tasks and two symptom rating blocks (cf. Figure 1). At the end of the exposure session, the participants used a Borg CR-100 scale to rate to what degree they believed the exposure to be harmful or beneficial for health. Similar to the valence ratings, participants added a plus sign before the rating if the exposure was judged as beneficial, and a minus sign before the rating if deemed harmful. After the exposure session, participants filled out the SCL-90 questionnaire. Participants were then debriefed and told about the different information biases.

### Statistical analysis

Analyses were performed using full factorial mixed model analyses of variance (ANOVAs) in IBM SPSS Statistics 20. The α was set at 0.05, with values < 0.1 considered as tendencies. Significant interaction effects were further analyzed and discussed only if they pertained to the factors Bias or Distress, as per the hypotheses. Effects not associated with the hypotheses are reported in Table 2. Greenhouse-Geisser correction was applied in cases where df > 1. In such cases, uncorrected *df*s are reported. Effect sizes are reported as eta sqared (η^2^) and were calculated using Microsoft Office Excel, 2010.

**Table 2:** ***F*-values (and, if statistically significant, eta-squared, η^2^) for the full factorial mixed model ANOVAs**.

	**Blank exposure**		**Chemical exposure**		**Symptom ratings**	**Cognitive performance**	
	**Intensity**	**Valence**	**Intensity**	**Valence**		**Plus/minus**	**Updating**
Time (T)	2.2	0.4	6.8 (0.13)^***^	0.4	25.1 (0.12)^***^	5.7 (0.04)^**^	16.9 (0.31)^***^
Bias (B)	7.2 (0.14)^*^	4.1(0.10)^†^	1.9	2.8	0.1	0.7	1.8
Distress (D)	6.2 (0.12)^*^	1.5	14.8 (0.28)^***^	8.5 (0.18)^**^	7.8 (0.17)^**^	1.9	4.0 (0.10)^‡^
T × B	0.3	0.2	1.2	0.1	1.5	1.2	0.5
T × D	1.3	2.4	2.9 (0.06)^*^	2.7 (0.07)^*^	4.2 (0.06)^*^	1.3	0.9
B × D	2.3	0.4	0.0	0.1	1.2	0.6	0.0
T × B × D	1.0	0.5	3.8 (0.07)^**^	1.3	0.4	1.6	0.2
Symptom (S)					18.3 (0.14)^***^		
T × S					6.3 (0.04)^***^		

No other significant effects involving the factors Symptom or Task (plus/minus lists); all F < 2.1.

* p < 0.05;

** p < 0.01;

*** p < 0.001;

† p = 0.052;

‡ p = 0.054.

## Results

### Manipulation of health risk perception

**Figure 2:**
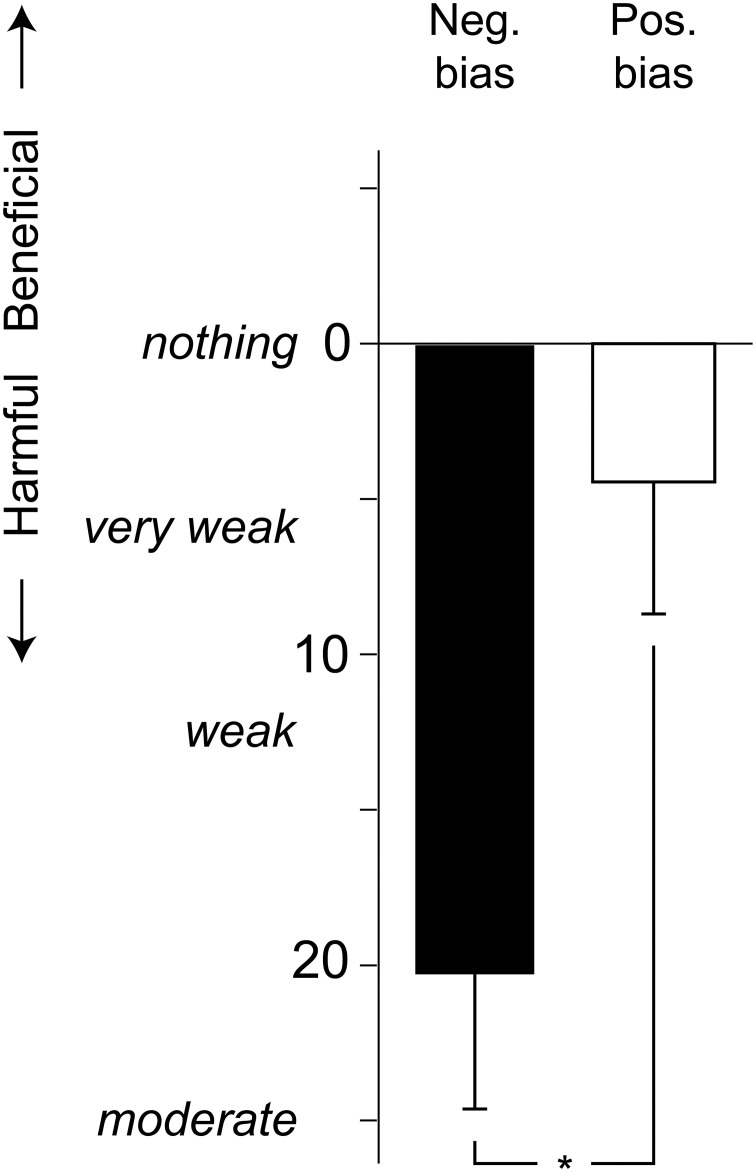
**Mean (+ standard error) ratings of harmful or beneficial health effects of the chemical exposure, using a Borg CR-100 scale.** *P*-values refer to the ANOVA parameter estimates (^*^p < 0.05).

The participants' judgments of beneficial or harmful health effects of the exposure was analyzed using a 2 × 2 (Bias [positive, negative] × Distress [low, high]) ANOVA. As seen in Figure 2, the negative bias group rated the exposure as more harmful than did the positive bias group, *F*_(1, 36)_ = 6.7, *p* = 0.014, η^2^ = 0.11. Distress did not affect the health risk judgments, *F*_(1, 36)_ = 0.8, *p* = 0.375.

### Intensity and valence ratings during blank exposure

**Figure 3:**
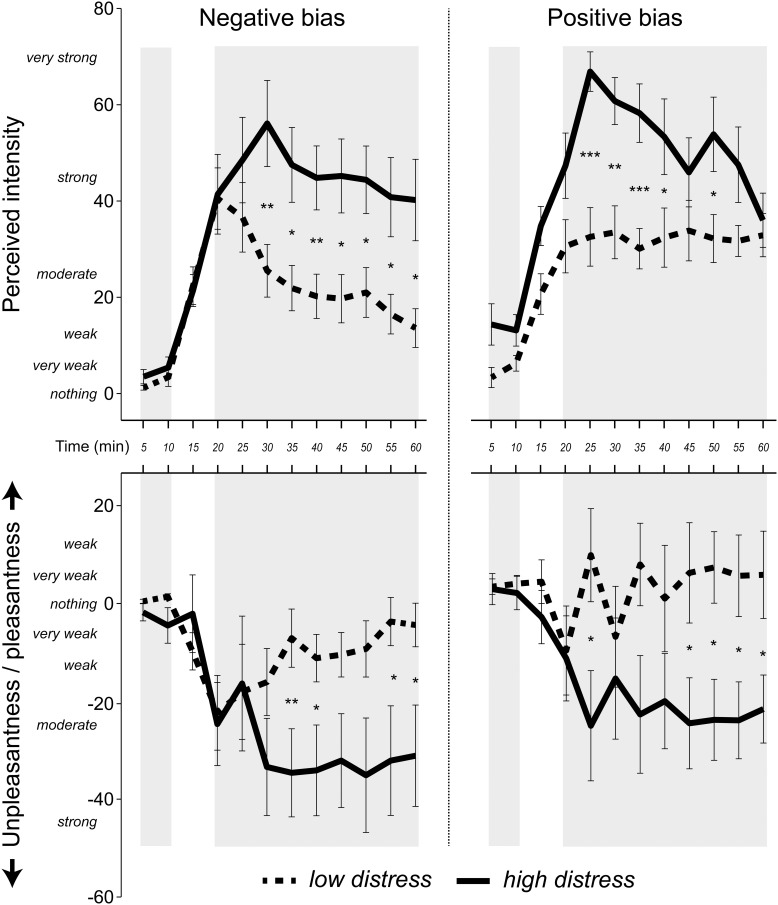
**Mean (± standard error) ratings of intensity and valence in 5 min intervals, using a Borg CR-100 scale.** Pleasantness was rated as positive values and unpleasantness as negative values. Shaded areas indicate values used in the statistical analyses. The first two ratings were made during blank exposure. The last nine ratings were made when the *n*-butanol concentration was at a stable concentration. *P*-values refer to the ANOVA parameter estimates (^*^p < 0.05, ^**^p < 0.01, ^***^p < 0.001).

Possible group differences of intensity ratings during blank exposure were investigated using a 2 × 2 × 2 (Time [first and second rating during blank exposure] × Bias [positive, negative] × Distress [low, high]) ANOVA. The positive bias group rated the blank exposure as more intense than did the negative bias group (cf. Figure 3) as seen by a main effect of Bias *F*_(1, 36)_ = 6.2, *p* = 0.017, η^2^ = 0.12. Additionally, the high distress group rated the blanks as more intense than the low distress group (cf. Figure 3) as seen by a main effect of Distress, *F*_(1, 36)_ = 7.2, *p* = 0.011, η^2^ = 0.14. An ANOVA with the same factors was performed on valence ratings, revealing a tendency of a main effect of Bias, *F*_(1, 36)_ = 4.1, *p* = 0.052, η^2^ = 0.10. The tendency is that the positive bias group rated the blanks as more pleasant than did the negative bias group (cf. Figure 3).

### Intensity and valence ratings during chemical exposure

A 9 × 2 × 2 (Time [nine ratings during chemical exposure] × Bias [positive, negative] × Distress [low, high]) ANOVA using intensity ratings during chemical exposure revealed a Time × Bias × Distress interaction, *F*_(8, 288)_ = 3.8, *p* = 0.007, η^2^ = 0.07. *Post-hoc* ANOVAs separating the factors Bias and Distress revealed a significant effect of Time for the low distress group receiving negative bias, *F*_(8, 288)_ = 5.5, *p* = 0.005, ?^2^ = 0.38, indicating that individuals in this group rated intensities as lower over time (cf. Figure 3). The high distress group receiving positive bias also reported lower intensities over time, as seen by a significant effect of Time, *F*_(8, 288)_ = 4.8, *p* = 0.009, η^2^ = 0.35 (cf. Figure 3). There was no effect of Time in the high distress group receiving negative bias, or the low distress group receiving positive bias (see Figure 3). Valence ratings were analyzed using an ANOVA with the same factors, yielding a Time × Distress interaction *F*_(8, 288)_ = 2.7, *p* = 0.048, η^2^ = 0.07. Figure 3 reveals that the ratings of the low distress group approached zero over time, whereas the valence ratings of the high distress group remained negative over time.

### Symptom ratings

**Figure 4:**
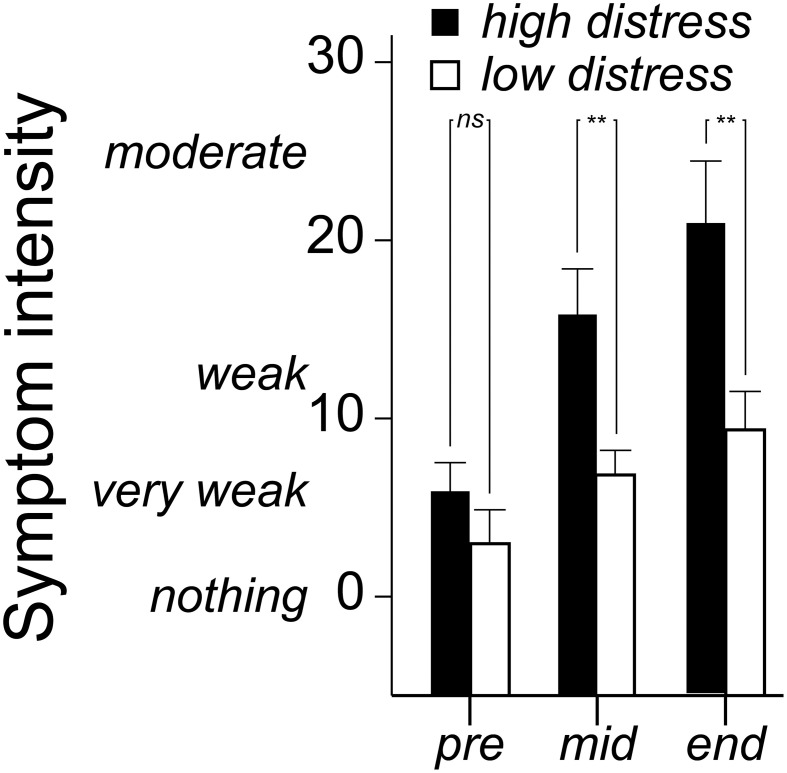
**Mean (+ standard error) ratings of eye irritation, nose irritation, skin irritation, throat irritation, shortness of breath, concentration difficulties, dizziness, tiredness, headache, and nausea before (pre), in the middle of (mid) and at the end of the *n*-butanol exposure session.** Ratings are made on a Borg CR-100 scale. *P*-values refer to the ANOVA parameter estimates (^**^p < 0.01).

Symptom ratings (mean of eye irritation, nose irritation, skin irritation, throat irritation, shortness of breath, concentration difficulties, dizziness, tiredness, headache and nausea) were analyzed with a 3 × 2 × 2 (Time [three occasions] × Bias [positive, negative] × Distress [low, high]) ANOVA. There was a Time × Distress interaction, *F*_(2, 72)_ = 4.2 *p* = 0.026, η^2^ = 0.06. As seen in Figure 4, the significant interaction refers to the high distress group reporting greater symptoms in the middle and end of the session. Notably, Bias did not affect symptom ratings (Table 2).

### Cognitive performance

**Figure 5:**
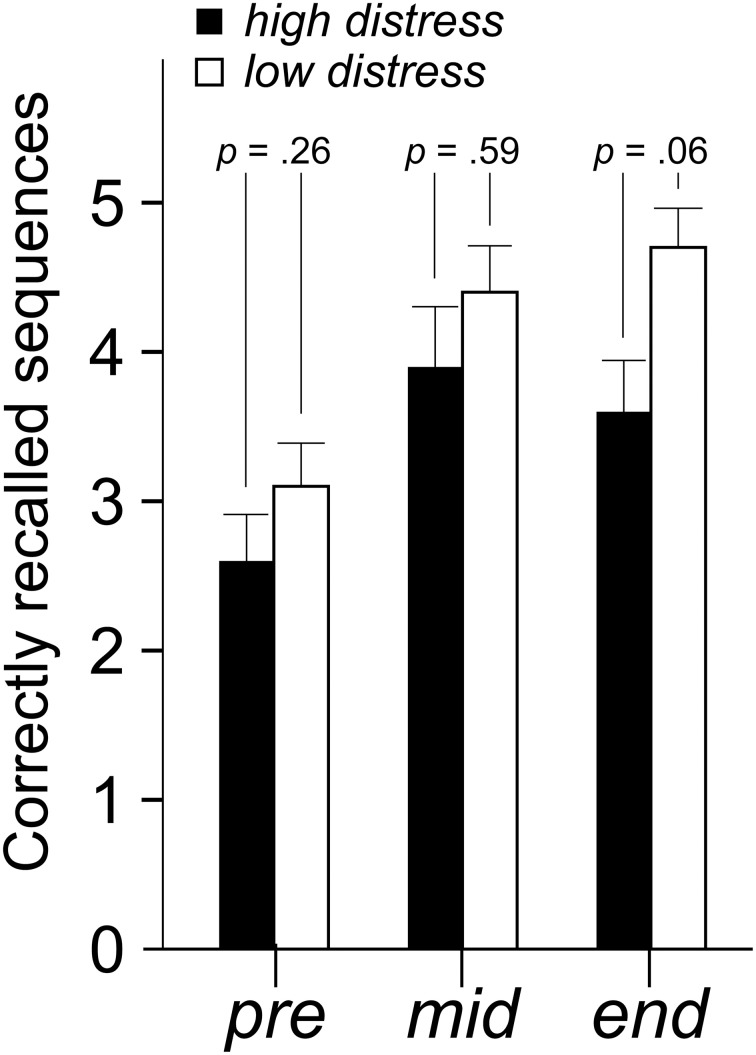
**Mean (+ standard error) number of correctly recalled sequences in the updating task.** *P*-values refer to the ANOVA parameter estimates.

Mean number of correct answers in the plus, minus and plus/minus lists were analyzed with a 9 × 2 × 2 (Time [nine blocks] × Bias [positive, negative] × Distress [low, high]) ANOVA. There were no significant effects for the factors Bias or Distress (Table 2). Number of correctly recalled sequences in the updating task was analyzed with a 3 × 2 × 2 (Time [three blocks] × Bias [positive, negative] × Distress [low, high]) ANOVA. There was a tendency of a main effect of Distress, with a lower amount of correctly recalled sequences in the high distress group *F*_(1, 36)_ = 4.0 *p* = 0.054, η^2^ = 0.10. Despite the lack of a Time × Distress interaction, parameter estimates nevertheless reveal that the Distress effect is greater at the end of the session. This is illustrated in Figure 5.

## Discussion

This study was conducted to investigate the effects of health-risk perception and self-reported distress on reactions to a low-level chemical exposure. Participants rated the perceived intensity and valence of blank stimuli and *n*-butanol, reported symptoms and performed cognitive tasks during the exposure session. Health-risk perception was manipulated by giving participants either positively or negatively phrased information regarding the compound used in the study. The manipulation was regarded as successful, as the participants receiving negative information judged the exposure to be more harmful compared with those receiving positive bias (Figure 2). Furthermore, participants were assigned into relatively high and low distress groups based on self-reports. Distress, in this regard, does not refer to pathological problems, but rather as normal variation in terms of rated well-being.

Our first hypothesis was that individuals reporting relatively high distress would sensitize to the chemical exposure described in a negative manner, whereas individuals reporting relatively low distress would habituate. Negative bias has previously been associated with increasing intensity ratings over time (Dalton, 1996, 1999). Similarly, traits such as anxiety have also been linked to higher perceived intensity of chemical exposure (Chen and Dalton, 2005). The analysis of the intensity ratings partly corroborated the first hypothesis by revealing an interaction between information bias, self-reported distress and time. The low distress group receiving negative bias reported intensities as decreasing over time to the invariant exposure (Figure 3). The high distress group receiving negative bias neither sensitized nor habituated to the exposure, but seemed to reach a stable plateau in terms of perceived intensity. Positive bias had the opposite effect on the rated intensities in the high and low distress group. Analyses of the Bias × Distress × Time interaction revealed that the high distress group habituated over time, whereas the low distress group did not (cf. Figure 3).

Among the interpretations of these results, we would like to point out one result in particular. By the end of the session in which participants received positive information bias, the high and low distress group rated the exposure as similar in terms of mean intensity (Figure 3). The same result was not seen when participants received negative information. The differences in perceived intensities between the high distress and low distress group rather increased with time. The mean perceived intensity of the distressed group was “strong” throughout the exposure session when rated according to the Borg CR-100 scale. The negatively biased non-distressed group rated the exposure as “weak” by the end of the session. These results suggest rather large, time-dependent discrepancies in basic sensory judgments between distressed and non-distressed individuals, but only when the exposure is deemed unhealthy. The interactions between bias and distress can be seen as an expansion of previous studies revealing a bias effect on intensity ratings (Dalton, 1996, 1999). The result may also be relevant for occupational exposure limits by revealing the extent of differences in the ratings of basic properties of the surroundings (Smeets and Dalton, 2005).

The analyses of valence and symptom ratings revealed effects of distress, but no interactions including information bias and distress in combination. The high distress group did not habituate in terms of rated unpleasantness, whereas the low distress group did. Moreover, the high distress group reported greater symptoms over time compared with the low distress group. These results do not contradict the first hypothesis stating that negative bias will have more deleterious effects in distressed individuals. However, as the same results were found when a positive bias was given, information bias seems to be redundant for these measures. The lack of a bias effect is seemingly at odds with earlier reports of bias effects on valence ratings (Kobayashi et al., 2007; Djordjevic et al., 2008; Nordin et al., 2013). There are, however, differences in exposure conditions that should be considered before regarding the current results as contradictory to previous studies. The long exposure may for instance hide initial bias differences in valence ratings. Although not part of the statistical analyses, the ratings in Figure 3 suggest possible bias effects in the beginning, but perhaps not at the end of the exposure. A hypothesis for future studies, based on this argument, would be that non-distressed individuals, to a greater degree than distressed, change their minds regarding the valence of extended exposures even if initially rating them as unpleasant.

The current study also revealed that the high distress group had a tendency of worse performance on the updating task, but not on plus/minus lists. In line with these results, trait anxiety has previously been associated with worse cognitive performance when exposed to chemicals, arguably due to greater distraction (Orbæk et al., 2005). The updating task used in the current study necessitates constant monitoring and updating of information in working memory (Miyake et al., 2000). A reasonable explanation for the worse performance in the high distress group is that the exposure, regarded as unpleasant and eliciting symptoms over time, interferes with this demanding task. The plus/minus lists are arguably less strenuous than the updating task, which might explain the lack of effects for this measure. Nordin et al. (2013) found a bias effect on plus/minus lists, a result that was not mirrored in the current study. The arithmetic task was, however, arguably easier in the current study, and consisted of adding and subtracting three from the presented number, instead of adding and subtracting seven as in the Nordin et al. study. The second hypothesis pertaining to worse performance in the high distress group is thus partly supported by current results.

Finally, the analyses revealed that the high distress group regarded the blank exposure as more intense than the low distressed group did. This may be seen as a higher false alarm rate in distressed individuals, parallel to that found in persons scoring high on somatization (Brown et al., 2012). Positive bias was also associated with higher intensity ratings of blanks, compared with the negative bias case. It is possible that this effect is due to the instructions, i.e., that the positive information referred to *n*-butanol as having a stimulating effect which may have been interpreted as being more intense. Moreover, there was a tendency of the positively biased group rating the blanks as more pleasant than did the negatively biased group, at least before the participants were exposed. Pleasantness is also the dimension that Knasko (1992) was able to manipulate by biased information during sham exposure.

Although investigated in a relatively small convenience sample calling for future replications, the tentative conclusion of this study is that traits, in this case self-reported distress, affects the reactions to low-level chemical exposure in terms of valence ratings, perceived symptoms and performance on a demanding cognitive task. Situational factors, i.e., health-risk perception interact with distress when making judgments of the intensity of the exposure. Relatively distressed individuals do not habituate in terms of intensity judgments when receiving negative information about an exposure, whereas relatively non-distressed individuals do. Generally, the lack of habituation in the distressed group could be seen as the first signs of the vicious cycle of responses that lead to the development of medically unexplained illnesses (Richardson and Engel, 2004; Deary et al., 2007; McEwen, 2007; Ganzel et al., 2010). Applied to e.g., occupational settings, the results could imply that individuals with normal sensory functioning, exposed to the same levels of ambient chemicals will over time differ significantly regarding how they experience their surroundings. A relatively non-distressed person will get used to the exposure. A relatively (albeit non-pathologically) distressed individual will perceive it as strong and unpleasant, as eliciting symptoms and affecting performance, especially if receiving negative information.

## Author contributions

All authors contributed to the design of the study and interpretation of results. Steven Nordin supervised the project. Anna-Sara Claeson prepared the exposure chamber and conducted the chemical analyses. Lisa Ledin, Frida Wisting, and Linus Andersson collected and analyzed the data. Linus Andersson wrote most of the manuscript.

### Conflict of interest statement

The authors declare that the research was conducted in the absence of any commercial or financial relationships that could be construed as a potential conflict of interest.
